# Draft Genome Sequence of a Multidrug-Resistant *Klebsiella pneumoniae* Strain Isolated from King Abdullah Medical City, Makkah, Saudi Arabia

**DOI:** 10.1128/genomeA.00375-16

**Published:** 2016-05-19

**Authors:** Rayd Algowaihi, Sami Ashgar, Bashir Sirag, Sheerin Shalam, Anmar Nassir, Abdalla Ahmed

**Affiliations:** a Microbiology Unit, Department of Laboratory and Blood Bank, King Abdullah Medical City, Makkah, Saudi Arabia; b Department of Microbiology, College of Medicine, Umm Al-Qura University, Makkah, Saudi Arabia; c Department of Surgery, College of Medicine, Umm Al-Qura University, Makkah, Saudi Arabia

## Abstract

Multidrug-resistant (MDR) Gram-negative infections represent a growing problem and a serious global threat. Carbapenem-resistant *Klebsiella pneumoniae* is perhaps cause the most difficult infection to treat and is associated with increased morbidity and mortality. Here, we report the draft genome sequence of an MDR *K. pneumoniae* strain isolated from Makkah, Saudi Arabia.

## GENOME ANNOUNCEMENT

*Klebsiella pneumoniae* is a multidrug-resistant (MDR) opportunistic pathogen with global prevalence (1). However, the situation in King Abdulla Medical City in Makkah, Saudi Arabia, is rather special. This tertiary-care hospital is located in the heart of Makkah City, where the Holy Pilgrimage of Islam draws millions of pilgrims every year. A large number of critically ill pilgrim patients are admitted to this hospital during the pilgrimage season. Therefore, clinical isolates from this hospital are quite diverse and expected to represent almost every part of the globe.

In this study, an MDR *Klebsiella pneumoniae* strain was isolated in 2015 from King Abdulla Medical City in Makkah, Saudi Arabia. The MDR *Klebsiella* isolate was recovered from a female patient with a urinary tract infection. Using phenotypic antimicrobial susceptibility testing methods, the strain was found to be resistant to amikacin, amoxicillin-clavulanic acid, ampicillin, cefazolin, cefepime, cefotaxime, cefoxitin, ceftazidime, cefuroxime, ciprofloxacin, ertapenem, fosfomycin, gentamicin, imipenem, levofloxacin, meropenem, mezlocillin, moxifloxacin, nitrofurantoin, norfloxacin, piperacillin-tazobactam, tetracycline, tigecycline, tobramycin, trimethoprim, and sulfamethoxazole. The only antibiotic to which it was susceptible was colistin.

The genome of the isolated MDR *Klebsiella pneumoniae* strain was sequenced using a 300-cycle paired-end library on Illumina MiSeq and resulted in 1,428,558 reads. A total of 1,113,884 reads were assembled into 210 contigs using SeqMan NGen version 12.3.1 (DNAStar, Madison, WI), with an average sequence coverage of 39× and average sequence quality of 32. The majority of the assembled contigs were >2,000 bp (92.1%), with *N*_50_ of 45 kb. The draft genome assembly of MDR *Klebsiella pneumoniae* is composed of 5,858,027 bp, with a G+C content of 56.75%. Genome annotation was performed by NCBI using the Prokaryotic Genome Annotation Pipeline and resulted in 5,889 protein-coding genes.

The assembled genome was used to predict the presence of acquired antibiotic resistance genes using the ResFinder server (2) and resulted in 19 hits, which include *aadA*, *aadA2*, *aph(3′)-VIa*, *armA*, ARR-3, *bla*_CTX-M-15_, *bla*_NDM-1_, *bla*_OXA-1_, *bla*_SHV-11_, *bla*_TEM-1A_, *catA1*, *cmlA1*, *dfrA1*, *dfrA12*, *fosA3*, *mph*(E), *msr*(E), *oqxB*, and *sul*.

### Nucleotide sequence accession numbers. 

This whole-genome shotgun project has been deposited at DDBJ/ENA/GenBank under the accession no. LSTN00000000. The version described in this paper is version LSTN01000000.
